# A prospective randomized controlled trial evaluating the safety and efficacy of patient blood management program in patients with gynecologic cancer (KGOG 4011/PBM)

**DOI:** 10.1136/ijgc-2023-004403

**Published:** 2023-04-24

**Authors:** Jeong-Yeol Park, Ok Ju Kang, Yoo-Young Lee, Young Seok Kim

**Affiliations:** 1 Department of Obstetrics and Gynecology, University of Ulsan College of Medicine, Asan Medical Center, Seoul, Korea (the Republic of); 2 Department of Obstetrics and Gyneoology, Samsung Medical Center, Sungkyunkwan University School of Medicine, Seoul, Korea (the Republic of); 3 Department of Radiation Oncology, University of Ulsan College of Medicine, Asan Medical Center, Seoul, Korea (the Republic of)

**Keywords:** Cervical Cancer, Uterine Cancer, Ovarian Cancer, Surgery

## Abstract

**Background:**

Gynecologic cancer has a high frequency of anemia, which is associated with increased morbidity and mortality. Blood transfusion is used to correct anemia, but carries its own side effects and problems in the blood supply have been emerging. As such, methods other than transfusion are needed to correct anemia in patients with cancer.

**Primary Objective:**

To determine whether intravenous administration of high-dose iron supplements before and after surgery as a patient blood management program is helpful in correcting anemia and reducing the frequency of transfusion in patients with gynecologic cancer.

**Study Hypothesis:**

Patient blood management will reduce the transfusion rate by up to 25%.

**Trial Design:**

This prospective, multicenter, interventional, randomized controlled study will consist of three steps. In step 1, the safety and effectiveness of patient blood management for surgical patients before, during, and after surgery will be evaluated. In steps 2 and 3, the safety and effectiveness of patient blood management in patients before, during, and after adjuvant radiation therapy and chemotherapy will be evaluated.

**Major Inclusion/Exclusion Criteria:**

Patients who are diagnosed with gynecologic cancer (ie, endometrial cancer, cervical cancer, ovarian cancer) and scheduled for surgery will be included and their iron deficiency status will be assessed. Only those with a pre-operative hemoglobin level of 7 g/dL or higher will be included. Patients who underwent neoadjuvant chemotherapy or pre-operative radiation therapy will be excluded. Also, patients with serum ferritin >800 ng/mL or transferrin saturation >50% on serum iron panel tests will be excluded.

**Primary Endpoint:**

Rate of transfusion within 3 weeks after surgery.

**Sample Size:**

Eligible participants will be randomly assigned in a 1:1 ratio (167 patients each) into the patient blood management group and the conventional management group.

**Estimated Dates for Completing Accrual and Presenting Results:**

Patient recruitment will be completed by mid-2025, and management and follow-up will be completed by the end of 2025.

**Trial Registration Number:**

NCT05669872.

## Introduction

Anemia is prevalent in cancer patients and affects 20–80% of patients with solid tumors.^1^ The causes of anemia in cancer patients include tumor-specific factors and treatment-related factors. Cancer-related chronic inflammation and cytokines can contribute to anemia by mediating the inhibition of erythropoietin production and iron sequestration.^2^ Acute bleeding from surgery, kidney failure due to chemotherapy, and bone marrow dysfunction due to chemotherapy and radiation or other treatments also contribute to cancer-related anemia.^3^ Most cases of anemia in cancer patients are iron deficiency anemia or anemia with functional iron deficiency.^4^ Anemia in cancer patients is associated with increased morbidity and mortality.^5 6^ Symptoms of anemia, which often manifest as shortness of breath, lethargy, palpitations, and fainting, can interfere with daily functioning and contribute to low quality of life.

Gynecologic cancer is associated with a high frequency of anemia (~85%). The frequency of anemia and blood transfusion is also reported to be very high during surgery, chemotherapy, and radiation therapy, which are the mainstay of treatment for gynecologic cancers.^7^ While the frequency of anemia before surgery ranges from 26% to 36%, the pre-operative and post-operative transfusion rates range from 41% to 77%.^8^ Previous studies have reported that anemia is associated with poorer outcomes for treatment and survival in gynecological cancers.^5 6^ Anemia is also associated with local tumor control failure, potentially related to treatment resistance by tumor hypoxia; accordingly, the hemoglobin level before treatment is a well-known prognostic factor of gynecologic malignancies.^5^ Therefore, the importance of anemia correction before treatment is increasingly emphasized in gynecologic oncology.

Blood transfusion was considered the only way to quickly correct anemia in the presence of acute blood loss; however, transfusion itself also carries side effects including transfusion-associated graft versus host disease, transfusion-transmitted diseases, alloimmunization to blood cell antigens, transfusion-related lung injury with pulmonary decompensation, and immunomodulation.^9^ Recently, problems in the supply of blood products for transfusion have emerged due to low birth rates, aging populations, and decrease in blood donations due to COVID-19.^10^ Therefore, the concept of patient blood management to replace blood transfusion and its necessity is being increasingly recognized. Patient blood management is defined as a patient-centered systematic evidence-based approach to improving patient outcomes by managing and preserving the patient’s own blood while promoting patient safety and empowerment.^11^ Patient blood management usually consists of three stages for patients undergoing surgery. Before surgery (stage 1), anemia is diagnosed and treated, and the red blood cell mass is maximized by improving the production of blood cells and supplying high-dose intravenous iron. During surgery (stage 2), hemostasis is optimized and blood loss is reduced through medical and surgical methods. After surgery (stage 3), the physiological reserve is optimized to prevent anemia. Through these stages, patient blood management can reduce the need for blood transfusions, cut associated healthcare costs, and stockpile blood components for those who need them the most.^12^

In a recent randomized controlled trial in adults with isovolumic anemia following radical gastrectomy, the use of intravenous ferric carboxy-maltose compared with placebo was more likely to result in an improved hemoglobin response at 12 weeks and led to significantly greater improvements in iron panels such as serum ferritin level and transferrin saturation levels.^13^ This method involving ferric carboxy-maltose can be applied to radiation therapy or chemotherapy for patients with cancer. However, there are no prospective studies that have evaluated the safety and efficacy of patient blood management for patients with gynecologic cancer undergoing surgery. Therefore, the aim of this study is to determine whether intravenous administration of high-dose iron supplements before and after surgery as a patient blood management program is helpful in correcting anemia and reducing the frequency of blood transfusion in patients with gynecologic cancer undergoing surgery. We also plan to determine whether the use of high-dose iron supplementation during adjuvant radiation (with or without chemotherapy) or adjuvant chemotherapy is helpful in the correction of anemia.

## Methods and Analysis

### Trial Design

This is a prospective, multicenter, interventional, comparative, randomized controlled trial involving a total of 15 institutions in South Korea, of which the Asan Medical Center (Seoul, South Korea) will serve as the leading institution. This study was approved by the institutional review board of Asan Medical Center (approval ID: 2022–1674, approval date December 6, 2022) and is registered in ClinicalTrial.gov (identifier number: NCT05669872). Informed consent will be obtained from all patients before participation.

**Figure 1:**
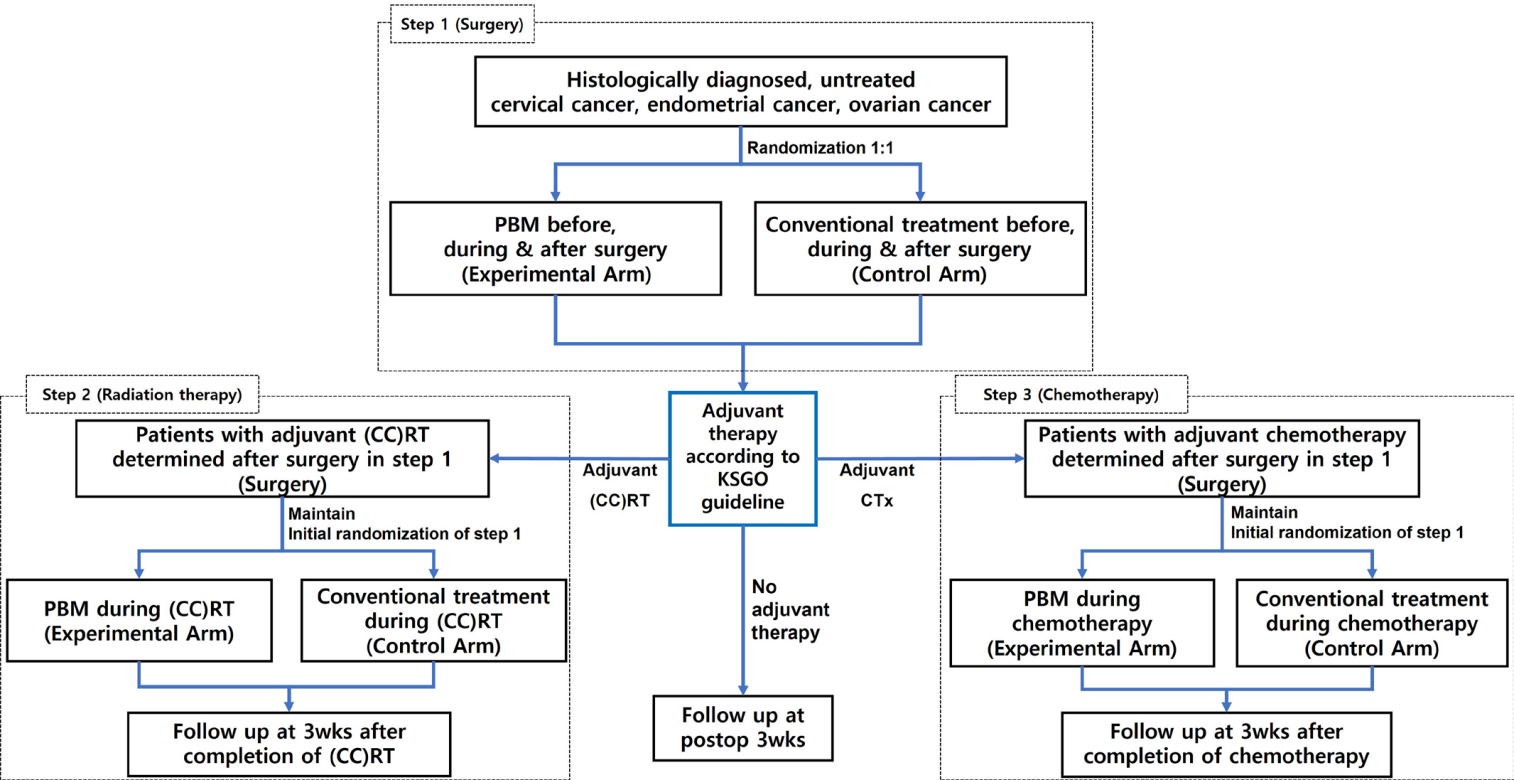
Study schema. (CC)RT, (concurrent chemo-) radiation therapy; KGSO, Korean Society of Gynecologic Oncology; PBM, patient blood management.

The study schema is shown in Figure 1. This study consists of three steps. In step 1, the safety and effectiveness of patient blood management for surgical patients before, during, and after surgery will be evaluated. In step 2, the safety and efficacy of patient blood management of patients before, during, and after adjuvant radiation therapy or concurrent chemoradiation therapy after surgery will be evaluated. In step 3, the safety and efficacy of patient blood management for patients before, during, and after adjuvant chemotherapy after surgery will be evaluated. Study treatments in each step are as follows.

#### Step 1: Anemia Correction in Surgery

**Figure 2:**
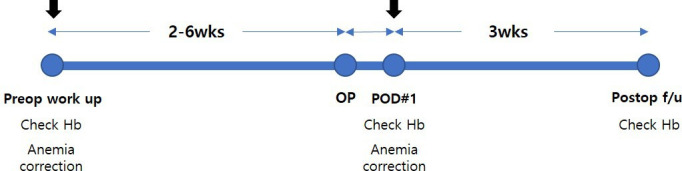
Examination and treatment of anemia in step 1 (surgery). Hb, hemoglobin; PBM, patient blood management; preop, preoperative; postop, postoperative; POD, postoperative day; wks, weeks.

Blood tests will be performed between 2 and 6 weeks before surgery and anemia will be corrected according to the test results for each group as follows. The treatment and examination schedule for step 1 is shown in Figure 2.

##### Patient Blood Management Group

If the hemoglobin level is between 7 g/dL and 12 g/dL in the preoperative blood test, 1000 mg of ferric carboxy-maltose will be administered between 2 and 6 weeks before surgery. During surgery, the need for transfusion will be decided according to the judgment of the attending surgeon and anesthesiologist in case of hemodynamic instability. A blood test will be performed the day after surgery, and if the hemoglobin level is between 7 g/dL and 12 g/dL, 1000 mg of ferric carboxy-maltose will be administered; if the hemoglobin level is 7 g/dL or less, two packs of red blood cells (RBC) will be transfused. The hemoglobin level will be re-evaluated at 3 weeks post-operatively.

##### Conventional Management Group

If the hemoglobin level is between 8 g/dL and 10 g/dL, one pack of RBC will be transfused and, if the hemoglobin level is 8 g/dL or less, two packs of RBC will be transfused. Erythropoietin and oral iron supplements will be allowed, but not intravenous iron supplements. During surgery, the need for transfusion will be decided according to the judgment of the operating surgeon and anesthesiologist in case of hemodynamic instability. A blood test will be performed the day after surgery, and if the hemoglobin level is between 8 g/dL and 10 g/dL, one pack of RBC will be transfused; if the hemoglobin level is 8 g/dL or less, two packs of RBC will be transfused. Post-operatively, erythropoietin and oral iron supplements will be allowed. The hemoglobin level will be re-evaluated 3 weeks post-operatively.

Surgical treatment and adjuvant treatment will be carried out according to the guidelines published by the Korean Society of Gynecological Oncology, which are consistent with the recent guidelines of the National Comprehensive Cancer Network. All surgery-related complications occurring during and after surgery during hospitalization will be evaluated using Dindo’s classification.^14^

#### Step 2: Anemia Correction in Adjuvant (Chemo)radiation Therapy

**Figure 3:**
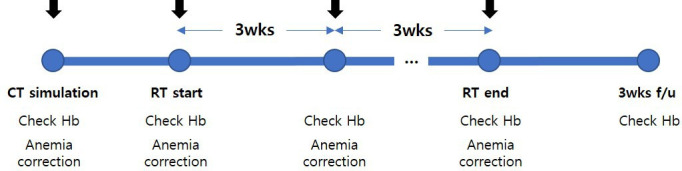
Examination and treatment of anemia in step 2 (radiation therapy). CT, computed tomography; Hb, hemoglobin; PBM, patient blood management; RT, radiation therapy; wks, weeks.

Anemia will be corrected after performing blood tests on the day of computed tomography (CT) simulation, during (concurrent chemo-) radiation therapy ((CC)RT), and every 3 weeks (visit window±1 week) after the end of treatment. The hemoglobin level will be re-evaluated at 3 weeks after the end of treatment. The treatment and examination schedule for step 2 is shown in Figure 3.

##### Patient Blood Management Group

If the hemoglobin level is between 7 g/dL and 12 g/dL in the pre-treatment blood test, 1000 mg of ferric carboxy-maltose will be administered; if the hemoglobin level is 7 g/dL or less, two packs of RBC will be transfused.

##### Conventional Management Group

If the hemoglobin level is between 8 g/dL and 10 g/dL, one pack of RBC will be transfused; if the hemoglobin level is 8 g/dL or less, two packs of RBC will be transfused. Erythropoietin and oral iron supplements will be allowed but not intravenous iron supplements.

#### Step 3: Anemia Correction in Adjuvant Chemotherapy

**Figure 4:**
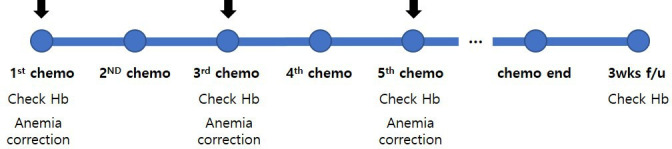
Examination and treatment of anemia in step 3 (chemotherapy). Chemo, chemotherapy; Hb, hemoglobin; PBM, patient blood management; wks, weeks.

Anemia will be assessed by performing blood tests the day before the start of chemotherapy and every two cycles during chemotherapy. The hemoglobin level will be re-evaluated at 3 weeks after the end of treatment. The treatment and examination schedule for step 3 is shown in Figure 4.

##### Patient Blood Management Group

If the hemoglobin level is between 8 g/dL and 12 g/dL in the pre-treatment blood test, 1000 mg of ferric carboxy-maltose will be administered; if the hemoglobin level is 8 g/dL or less, two packs of RBC will be transfused. Erythropoietin may be used in patients with a symptomatic hemoglobin level of 12 g/dL or less or an asymptomatic hemoglobin level of 8 g/dL or less.

##### Conventional Management Group

If the hemoglobin level is between 8 g/dL and 10 g/dL, one pack of RBC will be transfused; if the hemoglobin level is 8 g/dL or less, two packs of RBC will be transfused. Erythropoietin and oral iron supplements will be allowed but not intravenous iron supplements.

### Participants

#### Inclusion Criteria

Study patients must satisfy all of the following selection criteria: (1) women aged 20–80 years; (2) untreated histologically diagnosed cervical cancer, endometrial cancer, or ovarian cancer (including cases diagnosed by imaging without biopsy in the case of ovarian cancer); (3) Eastern Cooperative Oncology Group performance status 0–2; (4) American Society of Anesthesiologists physical status 1 or 2; (5) pre-operative hemoglobin ≥7 g/dL; (6) scheduled to undergo pre-operative iron panel test (serum ferritin, iron, total iron binding capacity, transferrin); (7) proper organ function with white blood cell count >3000/μL, platelet >100 000/μL, creatinine <2.0 mg/dL, bilirubin <1.5× normal, and serum glutamic-pyruvic transaminase, serum glutamic-oxaloacetic transaminase, and alkaline phosphatase ≤3× institutional upper limit normal; and (8) voluntarily signed the informed consent form.

#### Exclusion Criteria

The exclusion criteria are as follows: (1) unable to provide informed consent on their own; (2) allergic to existing iron preparations; (3) underwent neoadjuvant chemotherapy or pre-operative radiation therapy; (4) have had or received cancer treatment within 5 years, except for non-melanoma skin cancer, cervical intra-epithelial tumor, and superficial cancer of the stomach and bladder; (4) presence of iron overload or iron utilization disorders; and (5) serum ferritin >800 ng/mL or transferrin saturation >50% on iron panel tests.

### Endpoints

The primary endpoint is the rate of transfusion within 3 weeks after surgery. The secondary endpoints include transfusion rate within 3 weeks after radiation and chemotherapy, and the comparison of hemoglobin levels, frequency of anemia and blood transfusion before adjuvant therapy, frequency of delayed adjuvant therapy due to anemia, cost-effectiveness, quality of life, and adverse events after iron supplement treatment or blood transfusion.

### Sample Size

We performed sample size calculation through the Z-test with unpooled variance. Previous studies showed that the expected transfusion rate without patient blood management is 40%,^15 16^ and that the blood transfusion rate could be reduced by 25% through patient blood management. The following assumptions were made for calculating the required number of study participants: transfusion rate before, during, and after surgery when implementing patient blood management of 40%; level of significance α=0.05 (two-sided); and type II error, β=0.2 (ie, power=80%). The sample size was calculated using PASS version 15 (NCSS, Kaysville, Utah, USA) using the above settings and a total of 150 patients per group (total of 300 patients) were deemed necessary. Considering a dropout rate of 10%, we intend to enroll 167 patients per group (total 334 patients).

### Randomization and Blinding

Patients will be randomly assigned in a 1:1 ratio to the patient blood management group and the conventional management group. A randomization table will be prepared using SAS software (SAS Institute, Cary, North Carolina, USA) by an independent statistician and study patients will be assigned using the stratified block randomization method. Stratification will be performed according to the type of cancer, International Federation of Gynecology and Obstetrics stage, and methods of surgery. Due to the nature of the study, blinding is not possible.

### Statistical Methods

The main purpose of this study is to compare the transfusion rate according to the implementation of patient blood management in patients with gynecologic cancer undergoing surgery. For other safety endpoints such as laboratory tests and vital signs, continuous data will be presented using descriptive statistics (ie, mean, SD, median, min, max) for each group, and categorical variables will be presented using frequency and percentage. If necessary, the Student t-test or Wilcoxon rank sum test will be performed for continuous data to test for differences in other safety endpoints between groups, and the χ^2^ test or Fisher’s exact test will be performed for categorical variables. Statistical analysis will be performed using IBM SPSS Statistics for Windows, Version 26.0 (IBM Corp, Armonk, New York, USA).

## Discussion

Anemia in patients with cancer is associated with increased morbidity and mortality. Traditionally, blood transfusion has been performed to correct anemia in these patients, but transfusion has its own side effects and problems in blood supply have emerged recently. Therefore, many studies have been conducted to evaluate whether anemia can be effectively corrected using methods other than blood transfusion. In a phase III randomized study conducted at seven centers in South Korea, a placebo or ferric carboxy-maltose was administered to patients who had undergone extensive gastrectomy and had a hemoglobin of <7–10 g/dL after a few days of surgery.^13^ The hemoglobin levels were compared at 12 weeks after surgery and the results showed that the hemoglobin level was significantly higher in the iron supplement group by 2 g/dL or more.

However, there are no prospective studies that have evaluated the safety and efficacy of patient blood management for patients with gynecologic cancer undergoing surgery. Therefore, this trial is designed to demonstrate the safety and effectiveness of patient blood management in patients with gynecologic cancer undergoing surgery, adjuvant (chemo-)radiation therapy, and chemotherapy. In addition, there are no randomized controlled trials that have evaluated whether patient blood management can reduce the transfusion rate and improve surgical outcomes. This study will be the first randomized controlled trial to evaluate these issues. Through this study, we seek to provide evidence that patient blood management can effectively lower the transfusion rate of patients with gynecologic cancer. Since anemia and blood transfusion are associated with a poor survival prognosis in patients, developing a strategy to reduce transfusion during treatment would lead to improvements in the quality of treatment and increase the survival rate.^5^ In addition, the reduction in blood transfusion and transfusion complications can bring about economic effects through the reduction of medical costs and hospitalization. A reasonable and economical medical system can be established by enabling efficient pre- and post-surgery management. Furthermore, our results may pave the way to standardize blood management guidelines for patients with gynecologic cancer before, during, and after surgery and adjuvant treatment, and develop them as treatment guidelines.
